# Productive Propagation of Rift Valley Fever Phlebovirus Vaccine Strain MP-12 in *Rousettus aegyptiacus* Fruit Bats

**DOI:** 10.3390/v10120681

**Published:** 2018-11-30

**Authors:** Anne Balkema-Buschmann, Melanie Rissmann, Nils Kley, Reiner Ulrich, Martin Eiden, Martin H. Groschup

**Affiliations:** 1 Institute of Novel and Emerging Infectious Diseases, Friedrich-Loeffler-Institut, 17493 Greifswald-Insel Riems, Germany; anne.balkema-buschmann@fli.de (A.B.-B.); melanie.rissmann@fli.de (M.R.); nils.kley@fli.de (N.K.); martin.groschup@fli.de (M.H.G.); 2 Department of Experimental Animal Facilities and Biorisk Management, Friedrich-Loeffler-Institut, Südufer 10, 17493 Greifswald-Insel Riems, Germany; reiner.ulrich@fli.de

**Keywords:** Rift Valley fever phlebovirus, MP-12 vaccine strain, virus reservoir, *Rousettus aegyptiacus* bat

## Abstract

Rift Valley fever phlebovirus (RVFV), the causative agent of an emerging zoonotic disease in Africa and Arabia, can infect a variety of species, predominantly ruminants, camelids, and humans. While clinical symptoms are mostly absent in adult ruminants and camelids, RVFV infection may lead to a serious, sometimes fatal disease in humans. Virus transmissions between individuals and between species mainly occur through mosquito bites, but direct or even indirect contact with infectious materials may also result in infection. Although the main reservoir of the virus is not yet identified, small mammals such as rodents and bats may act as amplifying hosts. We therefore inoculated *Rousettus aegyptiacus* fruit bats that are abundant in northern Africa with the vaccine strain MP-12, in order to elucidate the general competence of this species for virus propagation and transmission. We were able to detect the RVFV genome in the spleen of each of these animals, and re-isolated the virus from the spleen and liver of some animals. Moreover, we were able to identify the Gc RVFV surface antigen in mild subacute multifocal necrotizing hepatic lesions of one bat which was sacrificed 7 days post exposure. These findings demonstrate that *Rousettus aegyptiacus* fruit bats can propagate RVFV.

## 1. Introduction

In the last decades, massive Rift Valley fever phlebovirus (RVFV) epidemics have caused considerable economic losses in livestock production and have also led to fatal infections in humans in Africa and Arabia. RVFV is an arbovirus (arthropod-borne) of the family *Phenuiviridae*, genus *Phlebovirus*. The genome of this negative stranded RNA virus comprises three segments, encoding the RNA-dependent RNA polymerase (L segment), the two surface proteins Gn and Gc as well as the nonstructural protein NSm (M segment), the nucleoprotein NP and a further nonstructural protein NSs (S-segment).

The virus is endemic throughout many sub-Saharan African countries and has repeatedly caused large outbreaks [1]. The major route of virus transmission to livestock is through bites mainly by *Aedes* spp. More than 30 additional mosquito species belonging to other genera have also been shown to be competent vectors for this virus [2]. Clinical signs in livestock vary between species and depend largely on the age of the infected animal. Most severe symptoms are seen in small ruminants, where so-called “abortion storms” may result in 100% fetal and neonatal losses [3]. Humans are most frequently infected by contact to viraemic animals. Symptoms may vary from mild flu-like conditions to meningoencephalitis, retinitis, or even hemorrhagic fever syndrome [4,5].

The use of live-attenuated vaccines such as Clone 13 or MP-12 has shown to be highly efficient in the protection against productive RVFV infections [6]. MP-12, which was generated by serial passage of the Egyptian ZH548 strain in the presence of the chemical mutagen 5-fluorouracil [7], is one of the best characterized RVFV vaccines. Its efficacy has been demonstrated in numerous studies in sheep, cattle, and macaques [8,9,10,11,12], and recently even a phase 2 clinical trial in humans was performed [13]. Nonetheless, this vaccine may still cause abortions or have teratogenic effects in sheep when used during the first trimester of pregnancy [14], and even virus replication was found in the liver of vaccinated animals [15,16].

Pteropodid bats of the order Chiroptera have recently been identified as reservoir hosts for a variety of viral agents that are capable of causing severe disease in humans and/or livestock animals [17,18]. Due to some of their basic biological characteristics, bats may propagate disease agents over extended periods of time and geographical areas [19,20]. Migratory species fly over long distances and therefore can efficiently spread infectious agents. Most bats live in colonies including enormous numbers of individuals, and their body temperatures may physiologically vary between less than 20 °C during hibernation and more than 40 °C during flight [21,22]. This variability in body temperatures may even have facilitated the evolution of viruses which are stable under different temperature conditions, and thus still display a high pathogenicity under febrile or cooler conditions in other mammals [20].

In recent years, human and wildlife habitats are increasingly overlapping due to the growth of the human population and conquest of human space, intensified agricultures, as well as ecological and climate changes. This results in considerably closer contact between humans, livestock, and wildlife such as bats and therefore in an increased risk of disease transmission [20,23]. It is therefore feasible that many viruses have been circulating unnoticed in wildlife for many generations but were recently discovered as pathogens after unprecedented spillover events.

*Rousettus aegyptiacus* fruit bats are abundant in the RVFV endemic areas in Africa and in the Middle East, as well as on Cyprus. They live in colonies of up to several thousand individuals [24]. Due to their close contact to human settlements, it has been speculated in the past that bats may act as reservoir hosts of RVFV [25,26]. First indications of bats playing a role in the virus ecology of RVFV were found 1984, when tissues of *Micropterus pusillus* and *Hipposideros abae* from Guinea were found to carry RVFV [27]. More RVFV detections were reported in Guinean bats in 1996 [28]. Recent in vitro approaches also found respiratory epithelial cells of the two bat species *Eidolon helvum* (Pteropodidae) and *Carollia perspicillata* (Phyllostomidae) to be highly susceptible for the attenuated Rift Valley fever Clone 13 virus [29]. The general susceptibility of the genus *Rousettus* to members of the order Bunyavirales has been documented by isolation of a novel bat phlebovirus from *Rousettus leschenaultii* [30] and a novel orthobunyavirus from bat fly ectoparasites of *Rousettus aegyptiacus* in South Africa [31]. Recently, antibodies against Crimean-Congo hemorrhagic virus (CCHFV) have been detected in *Rousettus aegyptiacus* species from Gabon, indicating infection with CCHF-like viruses that also belong to the Bunyavirales family [32]. Moreover, fruit bats have been shown to be competent amplifying hosts for flaviviruses. In this context there is some indication—based on blood meal analysis—that mosquitoes feed on bats [33].

Although several studies have highlighted the potential role of Chiroptera as reservoirs for RVFV, data regarding levels of viremia, seroconversion, or potential virus shedding upon infection are not available to date. We have therefore, in a small-scale pilot experiment, investigated the general competence of *Rousettus aegyptiacus* bats to function as RVFV reservoir hosts. This was done by subcutaneous inoculation of three animals with the attenuated RVFV vaccine strain MP-12, which is known to carry the same antigenic characteristics as the pathological strains.

## 2. Materials and Methods

### 2.1. Virus and Cell Culture

The MP-12 strain of RVFV was generously provided by the meanwhile deceased Richard Elliot, University of Glasgow, Centre for Virus Research, United Kingdom. MP-12 was propagated in Vero 76 cells (Collection of Cell Lines in Veterinary Medicine, Friedrich-Loeffler-Institut, Germany). The virus titer was determined using a 50% tissue culture infective dose (TCID_50_) assay, calculated as described by *Spearman* and *Kaerber*. Briefly, 100 μL of 10-fold serial diluted MP-12 were added to 90% confluent monolayers of Vero 76 cells. After incubation at 37 °C, 5% CO_2_ for 6 days, plates were fixed with neutral buffered formalin and stained with crystal violet.

### 2.2. RVFV MP12 Exposure of R. aegyptiacus Bats

**Figure 1:**
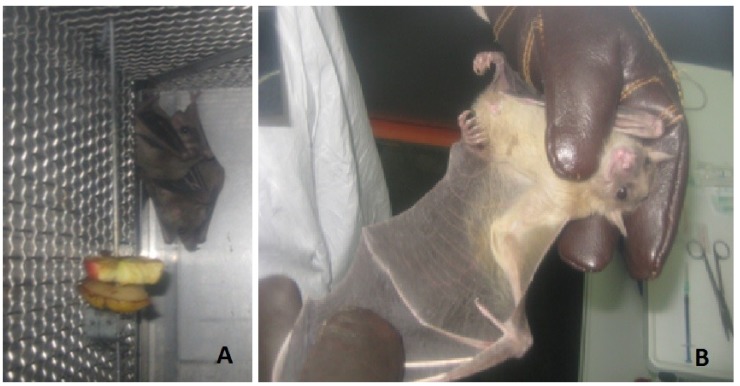
Handling of the bats during the immunization experiment. (**A**) Two bats kept together in one cage; (**B**) preparation of the bat for collection of blood sample. Due to the calm temperament of these animals, the sampling could be done without general anesthesia.

*R. aegyptiacus* bats were derived from the breeding colony at the Friedrich-Loeffler-Institut. For this study, four animals (two males, two females) were tagged individually with transponders and kept in cages (two bats per cage) as shown in Figure 1A,B. All staff entering the animal room wore personal protection equipment (PPE) adequate for the handling of biosafety level 3 (BSL-3) agents. In addition, thick leather gloves were worn when handling the bats. The animals were kept in a room at 24 °C and a humidity >55%. They were fed fresh fruits (apple, banana, pear, honey melon) ad libitum. Fresh water was available to them ad libitum. Three animals (ID 6210; ID 9988; ID 1740) were injected subcutaneously between the shoulder blades with 0.5 mL of a 10^6.125^ TCID_50_/mL virus suspension, while one animal (ID 8653) was mock-immunized with minimal essential medium (MEM) and 2% fetal calf serum (FCS), and kept as a negative control in contact with one of the immunized animals (ID1740, sacrificed at day 31 days post inoculation (dpi)). The virus suspension that was used for the immunization was eventually re-titrated on Vero 76 cells.

The animals were checked daily for behavioral or clinical anomalies. They were sampled immediately before the immunization (oral and rectal swabs, serum) and every third day throughout the experiment. Any urine or feces that were secreted during handling and sampling of the animals was also collected. Oral and rectal swabs were stored in MEM immediately after collection. At least 500 μL full blood (up to 800 μL) was taken to recover serum. Due to the calm temperament of these animals, the sampling could be done without general anesthesia.

The three MP-12-injected bats were sacrificed at 3 (ID 6210), 7 (ID 9988), and 31 dpi (ID 1740), while the mock-injected bat (ID 8653) was also kept for 31 days. For euthanasia, bats were first anesthetized using 100 mg/kg ketamine plus 5 mg/kg xylazine. When unconscious, 0.5 mL of a combination of embutramid, mebezonium, and tetracaine solution (T61; Intervet, Unterschleißheim, Germany) were administered intracardially.

Ethical approval for animal immunization at the Friedrich-Loeffler-Institut was provided by the competent authority (State Office for Agriculture, Food Safety and Fisheries Mecklenburg-Western Pomerania, LALLF 7221.3-2.5-004/10, approved at 14/4/2010) and the Ethics Committee of the Federal State of Mecklenburg-Western Pomerania, Germany, on the basis of national and European (RL 2010/63/EU) legislation.

### 2.3. Serology

All serum samples were tested with the ID Screen® RVFV competition multi-species ELISA (ID Vet, Montpellier, France) according to the manufacturer’s instructions. The ELISA is based on the nucleoprotein and antibody subtypes (IgG and IgM) are indistinguishable. Evaluation of samples was performed as suggested by the manufacturer. Samples containing RVFV antibodies, resulting in a percentage of inhibition lower than 40% were defined as “positive”. Samples giving readings of 40–50% were “inconclusive” and those above 50% were considered to be “negative”.

The serum neutralization test (SNT) was performed according to the Office International des Epizooties (OIE) Terrestrial Manual [34]. Briefly, 100 TCID_50_ of MP-12 was added to duplicates of heat inactivated sera in a 1:2 dilution. Following an incubation of 30 min at 37 °C and 5% CO_2_, 3 × 10^5^ Vero 76 cells were added to each well. Plates were incubated at 37 °C, 5% CO_2_ for 6 days. Neutralizing doses of 50% (ND_50_) were expressed as the reciprocal of the serum dilution that still inhibited >50% of cytopathogenic effect. Sera were diluted from 1:10 to 1:2560.

### 2.4. Detection of RVFV-Specific RNA

RNA was extracted from swab medium, serum, urine, and feces that were collected during the experiment, using the QIAamp^®^ Viral RNA Mini Kit (Qiagen, Hilden, Germany) according to the manufacturer’s recommendations. RNA of spleen, liver, kidney, bladder/urine, pancreas, large intestine, lung, heart, brain, and retina was extracted using the RNeasy Mini kit (Qiagen). As an internal extraction control, a MS2 bacteriophage was added to each sample [35]. The presence of RVFV-derived RNA was verified using a quantitative real-time RT-PCR (qRT-PCR) [3] As described earlier [15], the single internal mismatch of the applied forward primer with the MP-12 was shown to not significantly impact the detection. A synthetic RNA calibrator was utilized for quantification as described before [36].

### 2.5. Virus Isolation

Samples that tested positive in qRT-PCR were tested for the presence of functional MP-12 by inoculation of Vero 76 cells. About 30 mg of positive tissue was homogenized with 500 μL MEM with antibiotics. Fifty microliters of these tissue homogenates in 450 μL MEM with antibiotics (penicillin, streptomycin) and 2% FCS were added to 90% confluent Vero 76 cells. Flasks were incubated for 1 h at 37 °C, 5% CO_2_ with rocking every 15 min. Following adsorption, 2.5 mL MEM with 2% FCS and antibiotics were added. All flasks were incubated for a maximum period of 7 days with daily control for a cytopathogenic effect (cpe). RNA was isolated from harvested cell culture supernatants and a qRT-PCR was performed to validate the presence of MP-12.

### 2.6. Necropsy, Histopathology, and Immunohistochemistry

Necropsy was performed according to standard procedures under biosafety level 3 (BSL-3) conditions. Tissue specimens were fixed in 4% neutral buffered formaldehyde, processed, embedded in paraffin wax, sectioned at 2–4 μm thickness, and stained with hematoxylin and eosin. Immunohistology was performed using a mouse monoclonal antibody against the RVFV Gc-protein (clone: GC9A9) [37], the avidin–biotin–peroxidase complex method (ABC, Elite PK6100; Vector Laboratories, Burlingame, CA, USA) with 3-amino-9-ethylcarbazole (AEC, Dako, Glostrup, Denmark) as chromogen and hematoxylin counterstain.

Archival liver specimens from MP-12 RVFV strain-positive/negative alpacas and Vero 76 cell pellets from a previous study [15] were used as positive/negative controls. Furthermore, the primary antibody was replaced by tris-buffered saline on serial sections.

## 3. Results

### 3.1. Experimental Setup

Three *Rousettus aegyptiacus* bats were inoculated subcutaneously with RVF MP-12 strain (10^6.125^ TCID_50_) in order to raise antibodies as well as to investigate virus exposure effects. Animals were necropsied 3 days post inoculation (dpi) (ID 6210), 7 dpi (ID 9988), and 31 dpi (ID 1740). One animal (injected with mock inoculum) served as a negative control (ID 8653). All animals were checked daily for their health status, however, none of the animals showed any clinical symptoms throughout the experiment. At all times all bats were alert and did not show reduced appetite or any other behavioral abnormalities, as shown in Figure 1A,B.

### 3.2. Molecular Analysis

Oral and anal swabs, serum, as well as urine or fecal samples, were collected from all animals throughout the experiment and tested by qRT-PCR. Only one serum sample (ID 9988; collected 3 days post inoculation) gave a weak positive result in qRT-PCR with a *C*_t_-value of 38.25 and an estimated copy number of 9.5 × 10^1^, while all other serum samples collected during this study were negative, as shown in Table S1.

Interestingly RVFV RNA was also found in the spleen tissue of all three bats which had been inoculated with the MP-12 vaccine strain, as indicated by *C*_t_-values ranging between 31.11 (ID 6210), 31.26 (ID 9988), and 33.94 (ID 1740). The spleen sample of the mock-immunized control animal was negative. Furthermore, in the liver of one (ID 9988) of the three animals sacrificed at 7 dpi, RVFV-derived RNA was also recovered with a *C*_t_-value of 36.02. RVFV RNA was not detected in any of the other tissue samples of the four bats. Results and calculated copy numbers are summarized in Table 1.

In the case of positive qRT-PCR results (estimated copy numbers printed in red), the corresponding tissues were subsequently tested by virus isolation and immunohistochemistry (IHC). PCR negative tissues are not further analyzed.

### 3.3. Re-Isolation of MP-12 Virus

Spleen samples of all three exposed bats and the liver sample of ID 9988 were used for a virus isolation experiment on Vero 76 cells. Unfortunately, the volume of the qRT-PCR positive serum sample was insufficient to be able to address a virus isolation attempt. Cytopathogenic RVFV was demonstrated in the spleen of ID 9988 (sacrificed at 7 dpi) in this assay 5 days after inoculation of the cell culture. The cell culture supernatant was tested by qRT-PCR yielding a positive result (*C*_t_-value 17.52 with an estimated equivalent of 1.46 × 10^9^ copies/μL RNA). Additionally, a delayed cytopathogenic effect (cpe) was revealed after 7 days for the liver sample, indicating a very low virus load in this tissue. Eventually, this was also illustrated by the only very low positive qRT-PCR result for the corresponding tissue culture supernatant (*C*_t_-value 39.52; 5.66 × 10^1^ copies/μL RNA). The spleen sample of ID 6210 (sacrificed at 3 dpi) induced a mild cpe after 7 days of incubation, linked to a qRT-PCR *C*_t_-value of 35.3 and an estimated copy number of 1.78 × 10^3^/μL RNA. The cell culture supernatant of the RVFV re-isolation attempt from the spleen sample of ID 1740 (sacrificed at 31 dpi) was negative by qRT-PCR.

### 3.4. Serology

**Figure 2:**
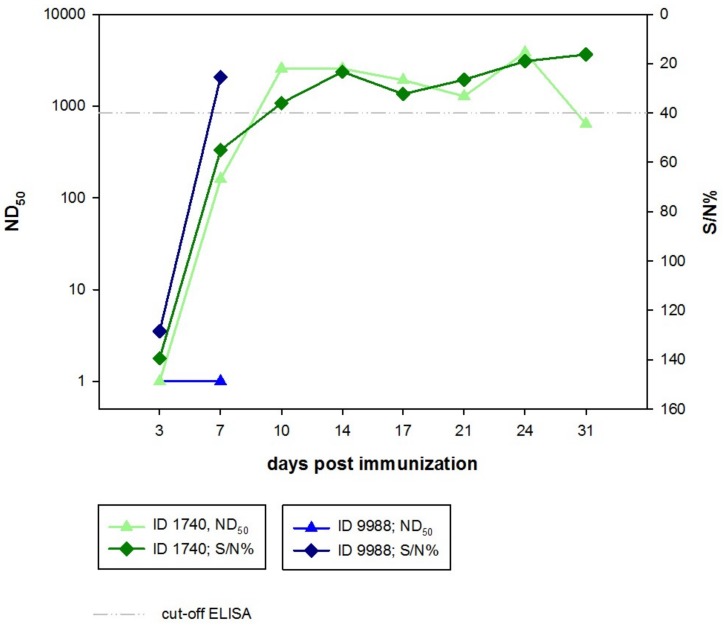
Serological reactivity of serum samples collected from ID 9988 sacrificed at 7 dpi, and ID 1740 sacrificed at 31 dpi. ND_50_: neutralizing doses of 50%; S/N%: percentage of inhibition.

The serum samples collected throughout the experiment as well as during the necropsies of the animals were tested using a commercial multi-species competition ELISA based on the detection of antibodies raised against the RVFV nucleoprotein, as shown in Figure 2. Unfortunately, there was no serum sample available from the animal sacrificed at 3 dpi. The serum of ID 9988 was negative at 3 dpi and positive at 7 dpi (day of necropsy) with a percentage of inhibition of 25.5%. Sera from the other animals (ID 1740) were still negative at 7 dpi with readings just below the cut-off value. However, all sera turned positive for all subsequent sampling dates until day 31 when the necropsy was carried out, displaying a percentage of inhibition as low as 16.29% at day 31.

The mock-immunized contact animal (ID 8653) remained negative, except for a single sample taken on day 24 which gave an inconclusive reading.

All serum samples were also tested by SNT to check for neutralizing antibodies. The serum samples of the animal sacrificed at 7 dpi were negative by SNT, although a positive ELISA result was obtained for the serum sample collected during the necropsy.

For the animal sacrificed at 31 dpi (ID 1740), positive SNT titers between 1:160 and 1:2560 were determined between day 7 and day 31 of the experiment, as shown in Figure 2. The mock-exposed contact animal did not develop neutralizing antibodies. Unfortunately, no serum was available from this animal for 21 days post immunization.

Neutralizing doses of 50% (ND_50_) (triangle) and the percentage of inhibition (S/N%) (diamond) detected in the ID Screen^®^ RVFV competition multi-species ELISA (ID Vet, Montpellier, France) are depicted for ID 1740 (green) and ID 9988 (blue).

### 3.5. Pathology

**Figure 3:**
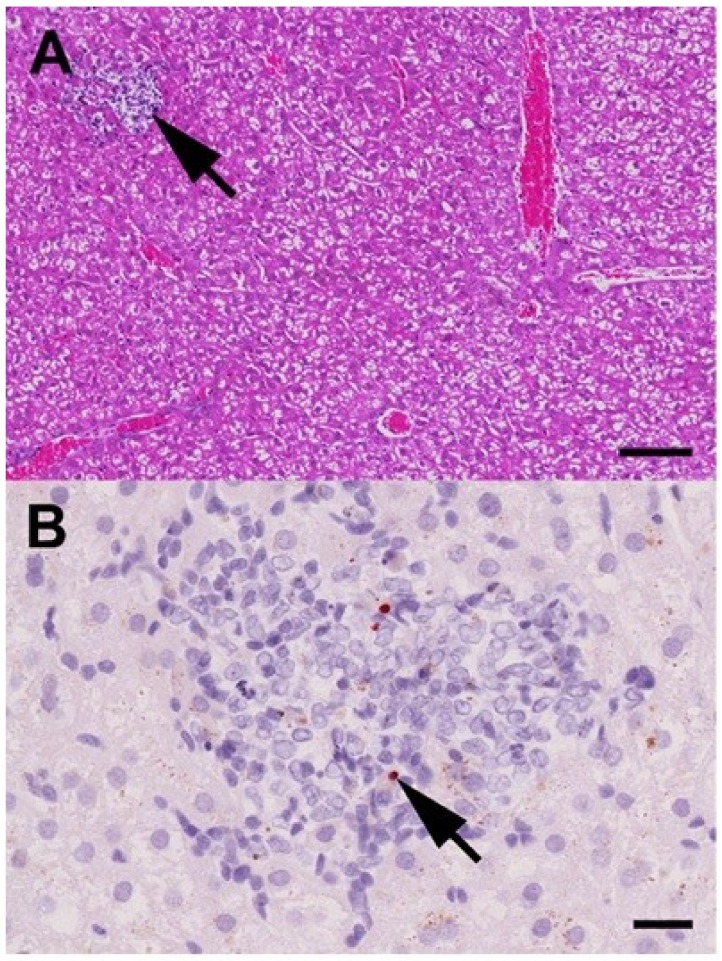
Histopathological findings in the liver of a *Rousettus aegyptiacus* fruit bat immunized with the MP-12 vaccine strain at day seven post immunization. (**A**) Histopathology shows few, randomly distributed foci of hepatocellular necrosis and loss with macrophage and lymphocyte infiltration (arrow). Furthermore, the hepatocytes display moderate, coalescing to diffuse, floccular cytoplasmic vacuolization, interpreted as a species-specific, relatively high level of glycogen storage. Hematoxylin-eosin. Bar = 100 μm; (**B**) Immunohistochemistry for Rift Valley fever phlebovirus (RVFV) Gc antigen reveals minor amounts of intra- and extracellular, strongly immunoreactive granula within the lesions (arrow), interpreted as debris remaining after virus-induced hepatocellular death. Immunohistochemistry, monoclonal mouse anti-RVFV Gc-protein antibody, avidin-biotin-peroxidase-complex method, 3-amino-9-ethyl-carbazol chromogen (red), hematoxylin counterstain (blue). Bar = 20 μm.

Necropsy of all four bats showed no gross lesions characteristic for RVF. Histopathology revealed a mild, subacute, multifocal, randomized, necrotizing hepatitis with macrophage and lymphocyte infiltration in ID 9988 (sacrificed at 7 dpi) as shown in Figure 3A, and a moderate, subacute, multifocal, periportal, lymphohistioplasmacytic and less frequently randomized, necrotizing hepatitis in ID 1740 (sacrificed at 31 dpi). Furthermore, the livers of all four bats displayed a variable degree of coalescing to diffuse hepatocellular cytoplasmic feathery vacuolization due to the accumulation of a Periodic acid–Schiff (PAS)-positive substance, interpreted as a species-specific, relatively high level of glycogen storage. Other findings, unrelated to the RVFV infection, included a bronchioloalveolar adenoma in the lung and a hepatocellular adenoma in the liver of ID 9988 (sacrificed at 7 dpi).

Immunohistochemistry revealed minor amounts of intra- and extracellular, strongly RVFV Gc-protein-positive granula within the necrotizing liver lesions in ID 9988 (sacrificed at 7 dpi), as shown in Figure 3B, interpreted as debris remaining after virus-induced hepatocellular death. No RVFV Gc-protein was detectable in the other examined organs (heart, lung, spleen, and kidney) of this and in none of the organs of the other three bats. 

## 4. Discussion

This pilot study provides a first molecular and serological indication for fruit bats representing a potential reservoir for Rift Valley fever virus. With an initial intention to produce bat-derived reference sera to RVFV, we have injected *Rousettus aegyptiacus* fruit bats subcutaneously with the attenuated life RVFV vaccine strain MP-12. No clinical symptoms were observed in these animals, however, there was clear evidence of virus replication following such an exposure. The here described study therefore indicates that *Rousettus aegyptiacus* fruit bats can be productively infected, even when only the attenuated RVFV vaccine strain (MP-12) is used. This raises the presumption that fruit bats are generally susceptible to RVFV which shall be verified in a follow-up study in the same bat species, using a non-attenuated RVFV strain. Although small ruminants have been identified as the major livestock host species for RVFV, the wildlife reservoir host species for this agent is yet to be determined [26].

First of all, we found clear evidence of virus replication in all three inoculated animals since RVFV-associated RNA was detected in the spleens. Although the viral load was rather low with values between 2.6 × 10^1^ and 1.9 × 10^3^ copies/μL, these results clearly prove virus propagation. In addition, one individual (ID 9988) was viraemic at 3 dpi and viral RNA was also detected in the liver at 7 dpi. The presence of RVFV Gc antigen was also demonstrated by immunohistochemistry within the subacute necrotizing liver lesions, which is a clear indication of virus propagation in this tissue. Virus-positive bat organs have already been detected in a single field study where RVFV could be isolated from organs of different bat species (*Micropteropus pusillus*, *Hipposideros abae,* and *Hipposideros caffer*) inoculated to suckling mice [27].

Bat ID 1740 developed neutralizing antibodies with titers between 1:160 and 1:1920 from day 7 onwards until the end of the experiment at 31 dpi, indicating a protective mechanism against RVFV replication. This is in accordance with recent findings of neutralizing antibodies in *Rousettus aegyptiacus* bats as well as minor epauletted fruit bats (*Epomophorus minor*) from Uganda [38]. However, no virus shedding via excretions such as saliva, feces, or urine was observed using this vaccine strain. Likewise, none of the animals showed any clinical signs. It remains to be determined whether this relatively mild course of the infection in this fruit bat is also true for infections with highly virulent RVFV strains. If this would be the case, infected fruit bats would be able to move around and spread the virus within colonies and be an infectious source for mosquitos. Perhaps such animals may even shed highly virulent RVFV efficiently, thereby facilitating a productive virus circulation in bat colonies.

To our knowledge, this is the first experimental study on the susceptibility of *Rousettus aegyptiacus* bats for RVFV. Only one related experiment has been performed, where a virulent RVFV strain was used to inoculate one *Miniopterus schreibersii* and three *Eptesicus capensis* bats by the oral or intramuscular route [25]. RVFV antigen could be detected in the liver and urine of one *M. schreibersii* bat, as well as in the brown fat of one *E. capensis* bat. Equal to our study, none of the bats showed clinical signs of infection. However, the absence of serological and molecular examinations rules out any further comparison of both studies.

Taken together, this pilot study proves the general susceptibility of *Rousettus aegyptiacus* fruit bats for the MP-12 RVFV vaccine strain. The here described work was intended as a technical feasibility study and aimed at determining the general susceptibility of *Rousettus aegyptiacus* fruit bats. Given their observed susceptibility even to a RVF vaccine strain, a follow-up study including larger animal numbers to be challenged with a virulent RVF strain under experimental high containment BSL-3 conditions will be addressed.

## Figures and Tables

**Table 1:** Results of qRT-PCR analysis of tissues from MP-12 inoculated *Rousettus aegyptiacus* fruit bats. dpi: days post inoculation; IHC: immunohistochemistry; n.d.: not done.

	**ID 6210 (3 dpi)**			**ID 9988 (7 dpi)**		
**Sample Type**	**PCR (Copies/μL)**	**Virus Isolation**	**IHC_**	**PCR (Copies/μL)**	**Virus Isolation**	**IHC_**
spleen	3.6 × 10^2^	+	neg	1.9 × 10^3^	+	neg
liver	neg	n.d.	n.d.	2.6 × 10^1^	+	pos
kidney	neg	n.d.	n.d.	neg	n.d.	n.d.
urine bladder	neg	n.d.	n.d.	neg	n.d.	n.d.
pancreas	neg	n.d.	n.d.	neg	n.d.	n.d.
small intestine	neg	n.d.	n.d.	neg	n.d.	n.d.
lung	neg	n.d.	n.d.	neg	n.d.	n.d.
heart	neg	n.d.	n.d.	neg	n.d.	n.d.
brain	neg	n.d.	n.d.	neg	n.d.	n.d.
eye	neg	n.d.	n.d.	neg	n.d.	n.d.
	**ID 1740 (31 dpi)**			**ID 8653 (31 dpi) Mock Control**		
**Sample Type**	**PCR (Copies/μL)**	**Virus Isolation**	**IHC_**	**PCR (Copies/μL)**	**Virus Isolation**	**IHC_**
spleen	5.9 × 10^2^	neg	neg	neg	n.d.	n.d.
liver	neg	n.d.	n.d.	neg	n.d.	n.d.
kidney	neg	n.d.	n.d.	neg	n.d.	n.d.
urine bladder	neg	n.d.	n.d.	neg	n.d.	n.d.
pancreas	neg	n.d.	n.d.	neg	n.d.	n.d.
small intestine	neg	n.d.	n.d.	neg	n.d.	n.d.
lung	neg	n.d.	n.d.	neg	n.d.	n.d.
heart	neg	n.d.	n.d.	neg	n.d.	n.d.
brain	neg	n.d.	n.d.	neg	n.d.	n.d.
eye	neg	n.d.	n.d.	neg	n.d.	n.d.

## Supplementary Materials

The following are available online at http://www.mdpi.com/1999-4915/10/12/681/s1, Table S1: Serum and swab samples tested by qRT-PCR. Viral load is specified in copies/µL (cop/µL).
