# Supplementary material for: Productive Propagation of Rift Valley Fever Phlebovirus Vaccine Strain MP-12 in Rousettus aegyptiacus Fruit Bats
Source: Viruses. 2018 Nov 30;10(12):681. doi: 10.3390/v10120681 (PMC6315703; doi:10.3390/v10120681)
Supplement: Supplementary file 1 [file viruses-10-00681-s001.pdf]

Animal	Sample	Days post inoculation (dpi)										
		0	3	6	9	12	15	18	21	24	27	30
ID 6210	serum	**neg**	***n.a.***									
	anal swab	**neg**	**neg**									
	oral swab	**neg**	**neg**									
ID 9988	serum	**neg**	**95 cop/µl**	**neg**								
	anal swab	**neg**	**neg**	**neg**								
	oral swab	**neg**	**neg**	**neg**								
ID 1740	serum	**neg**	**neg**	**neg**	**neg**	**neg**	**neg**	**neg**	**neg**	**neg**	**neg**	**neg**
	anal swab	**neg**	**neg**	**neg**	**neg**	**neg**	**neg**	**neg**	***n.a.***	**neg**	**neg**	**neg**
	oral swab	**neg**	**neg**	**neg**	**neg**	**neg**	**neg**	**neg**	***n.a.***	**neg**	**neg**	**neg**
ID 8653 (mock)	serum	**neg**	**neg**	**neg**	**neg**	**neg**	**neg**	**neg**	**neg**	**neg**	**neg**	**neg**
	anal swab	**neg**	**neg**	**neg**	**neg**	**neg**	**neg**	**neg**	***n.a.***	**neg**	**neg**	**neg**
	oral swab	**neg**	**neg**	**neg**	**neg**	**neg**	**neg**	**neg**	***n.a.***	**neg**	**neg**	**neg**

Supplementary Table S1: Serum and swab samples tested by qRT-PCR. Viral load is specified in copies/µl (cop/µl)

n.a. not available
